# Genetic Variability of Human Respiratory Syncytial Virus A Strains Circulating in Ontario: A Novel Genotype with a 72 Nucleotide G Gene Duplication

**DOI:** 10.1371/journal.pone.0032807

**Published:** 2012-03-28

**Authors:** AliReza Eshaghi, Venkata R. Duvvuri, Rachel Lai, Jeya T. Nadarajah, Aimin Li, Samir N. Patel, Donald E. Low, Jonathan B. Gubbay

**Affiliations:** 1 Ontario Agency for Health Protection and Promotion, Toronto, Ontario, Canada; 2 Mount Sinai Hospital, Toronto, Ontario, Canada; 3 University of Toronto, Toronto, Ontario, Canada; 4 The Hospital for Sick Children, Toronto, Ontario, Canada; Centers for Disease Control and Prevention, United States of America

## Abstract

Human respiratory syncytial virus (HRSV) is the main cause of acute lower respiratory infections in children under 2 years of age and causes repeated infections throughout life. We investigated the genetic variability of RSV-A circulating in Ontario during 2010–2011 winter season by sequencing and phylogenetic analysis of the G glycoprotein gene.

Among the 201 consecutive RSV isolates studied, RSV-A (55.7%) was more commonly observed than RSV-B (42.3%). 59.8% and 90.1% of RSV-A infections were among children ≤12 months and ≤5 years old, respectively. On phylogenetic analysis of the second hypervariable region of the 112 RSV-A strains, 110 (98.2%) clustered within or adjacent to the NA1 genotype; two isolates were GA5 genotype. Eleven (10%) NA1-related isolates clustered together phylogenetically as a novel RSV-A genotype, named ON1, containing a 72 nucleotide duplication in the C-terminal region of the attachment (G) glycoprotein. The predicted polypeptide is lengthened by 24 amino acids and includes a23 amino acid duplication. Using RNA secondary structural software, a possible mechanism of duplication occurrence was derived. The 23 amino acid ON1 G gene duplication results in a repeat of 7 potential O-glycosylation sites including three O-linked sugar acceptors at residues 270, 275, and 283. Using Phylogenetic Analysis by Maximum Likelihood analysis, a total of 19 positively selected sites were observed among Ontario NA1 isolates; six were found to be codons which reverted to the previous state observed in the prototype RSV-A2 strain. The tendency of codon regression in the G-ectodomain may infer a decreased avidity of antibody to the current circulating strains. Further work is needed to document and further understand the emergence, virulence, pathogenicity and transmissibility of this novel RSV-A genotype with a72 nucleotide G gene duplication.

## Introduction

Human Respiratory Syncytial virus (RSV) is the major cause of lower respiratory tract infection (LRTI) in infants and young children, and is also responsible for a significant proportion of RTIs in the elderly. It causes repeated infections throughout life due to limited immune protection from earlier RSV exposure [1], [2], [3]. RSV, classified in the Pneumovirus genus of the *Paramyxoviridae* family, is an enveloped virus with a negative-sense single-stranded RNA genome which encodes for 11 proteins. Two groups, RSV-A and RSV-B, have been described on the basis of reactions with monoclonal antibodies against the G and F glycoproteins [4], [5] and molecular differences of several genes [3]. Being major surface glycoproteins, G and F are mainly involved in virus attachment to cell receptors and mediation of cell membrane fusion, respectively [6], [7]. Hence, both proteins are highly accessible to neutralizing antibodies, with resultant accumulation of mutations in response to host immunological pressure [8].

RSV-A and RSV-B evolved separately at different time periods [5]. They co-circulate and both are responsible for epidemics, which are more commonly caused by RSV-A [9]. Genotyping of RSV-A and RSV-B viruses is based on the sequence variability of the G protein gene. Ten RSV-A genotypes have been reported from different geographical regions, and designated as GA1 to GA7 [10], [11], SAA1 (South Africa, A1) [12] and most recently, NA1 and NA2 [13]. RSV-B genotypes include GB1 to GB4 [10], SAB1 to SAB3 [12], and BA1 to BA6 (Buenos Aires) [14]. Interestingly, strains belonging to the BA genotype of RSV-B from Argentina exhibited a 60 nucleotide (nt) duplication in the second variable region of the G protein gene but have not caused any major outbreaks or been associated with serious clinical manifestations [15], [16], [17]. Genetic variability between RSV strains is a signature characteristic that may alter the pathogenicity and fitness of the virus, and contribute to the ability to cause repeated infections and outbreaks by immune system evasion.

The mature G glycoprotein consists of three unique regions consisting of the cytoplasmic tail (amino acids [AAs] 1–38), transmembrane domain (AA 38–66), and the ectodomain (AA 66–298). The C-terminal ectodomain of G protein is comprised of 2 variable regions flanking the putative receptor binding site, a conserved region of 13 AAs (AA 164–176) situated between them. Although the G protein is highly glycosylated with N- and O-linked sugars, these positions are poorly conserved [18]. The two variable regions of the ectodomain contain high serine and threonine residues, which are potential acceptor sites for O-linked sugars. These N-and O-linked oligosaccharides contribute to the antigenic structure of the G protein as well as impacting on virus infectivity [19], [20].

In this study we evaluated the genetic variability in the G protein gene of RSV-A viruses isolated from clinical samples collected in Ontario, Canada. Phylogenetic analysis was performed to establish the relationships between Ontario's strains and previously described RSV-A genotypes deposited in Genbank. In depth positive selection pressure analysis was also done to examine the replacement behavioural patterns of G protein gene encoded AAs. Further, we tried to derive a possible mechanism for the occurrence of an observed G gene duplication by viral RNA secondary structure analysis.

## Materials and Methods

### Ethics Statement

This study was considered exempt from University of Toronto's Health Sciences Research Ethics Board review as it involved deidentified respiratory tract samples that were tested as part of a clinical virology service provided by Public Health Ontario Laboratories. All test-positive samples and a proportion of test-negative samples are stored for possible further laboratory-based surveillance work. Samples and isolates included in this study were analyzed as part of the routine respiratory viral molecular surveillance program that supports Ontario's Ministry of Health and Long-Term Care.

### Specimen collection and viral isolates

Public Health Ontario performs a large proportion of primary respiratory viral testing for the province of Ontario from a variety of clinical settings including ambulatory, hospital and outbreaks. All consecutive HRSV culture isolates, identified from November 2010 to February 2011 at Public Health Ontario Laboratory – Toronto (PHOL), were selected for this study. . Following the testing algorithm for respiratory specimens, nasopharyngeal swabs were forwarded directly to PHL for respiratory viral testing. All nasopharyngeal swabs (NPS) from ambulatory and hospitalized, non-ICU patients are cultured for virus isolation in two cell lines, 1. either rhesus monkey kidney (RMK) or African green monkey kidney cells (AGMK), along with 2. WI-38 human embryonic lung fibroblast (Diagnostic Hybrids, Inc, Ohio, USA). Cell lines showing cytopathic effect are stained with a blend of murine monoclonal antibodies (MAbs) directed against seven respiratory viruses plus separate DFA Reagents, each consisting of MAb blends directed against a single respiratory virus, including RSV (D3 Ultra™ DFA Respiratory Virus Screening & ID Kit, Diagnostics Hybrids, Ohio, USA). In addition to viral culture, all NPS from infants under 12 months of age with bronchiolitis or pneumonia, and when requested in children ≤5 years of age, are initially screened by a rapid RSV antigen test (BinaxNOW® RSV kit, Binax Inc., Maine, USA). Samples submitted from patients in the outbreak or intensive care unit (ICU) setting undergo multiplex molecular testing for respiratory viruses, but not viral culture, and were not evaluated in this study

### RNA extraction and sub-grouping

Total nucleic acid was extracted from 250 ul of the supernatant of each cell-cultured sample using the NucliSens easyMAG extraction system (bioMérieux Canada Inc. Québec, Canada) according to manufacturer's instructions. Sub-grouping was undertaken targeting the nucleocapsid (N) gene using a modified duplex version of a previously published method [21].

### RT-PCR and Sequencing

A 900-bp fragment of the G gene and a 500-bp fragment of the F gene of RSV-A was amplified with the OneStep RT-PCR kit (QIAGEN). Primer G267 corresponds to bases 247 to 267 in the G glycoprotein of the A2 strain (Genbank accession number M11486) and F164 primer complementary to bases 164 to 186 in the F protein [22]. Sanger sequencing of the PCR products was carried out with the same primer pair used for amplifications on the 3730×l DNA sequencer (Applied Biosystems) using the BigDye Terminator v3.1 cycle sequencing kit (Applied Biosystems). Alternatively, RSV-A-655F primer [23] was used to amplify the C-terminal half of the G protein gene when primer G267 did not yield a good sequence.

### Phylogenetic analysis

The nucleotide sequences of a fragment of the second hypervariable region of G glycoprotein gene (264 nucleotides corresponding to codon positions 210 to 298) from RSV-A isolates were determined and compared with those of reference strains representing different RSV-A genotypes deposited in Genbank. Sequence editing was performed using Vector NTI® *Express* Software (Life Technologies™, California, USA). Multiple sequence alignments of the 264 nucleotides in the second hypervariable region of G gene compared to available reference genotypes were performed by the ClustalW algorithm. Phylogenetic analyses using the neighbor-joining method, and the statistical significance of the tree topology tested by bootstrapping (1,000 replicates) were performed using the MEGA 5.05 software [24]. The evolutionary distances were derived using the Kimura-2 parameter method [25]. The phylogeny of the partial Fusion (F) gene sequences was also constructed.

### Selection pressure analysis

In order to understand the selection pressure at codon sites, we used the multiple aligned dataset of all Ontario G-gene (C-terminal hypervariable region) sequences including NA1 as a reference sequence and the maximum likelihood (ML) tree as input for the CODEML program of Phylogenetic Analysis by Maximum Likelihood (PAML 4.4 version) [26], [27]. The program PAML incorporates different codon-based substitution models that account for variable ω (non-synonymous/synonymous ratio, dN/dS) for each codon site. In this analysis, we used four different codon substitution models that account for neutral (M1a and M7) and positive (M2a and M8) selection. The model M1a estimates a class of negatively selected sites with proportion *p*_0_, with ω_0_ = 0, and the remaining sites with proportion *p*_1_ (*p*_1_ = 1−*p*_0_), assuming ω_1_ = 1. The M2a model facilitates detection of an extra class of sites under positive selection with proportion *p*_2_ (where *p*_2_ = 1−*p*_1_−*p*_0_) with ω_1_>1. The model M7 incorporates a beta distribution (with parameters *p* and *q*) to account for variable ω among neutral or negatively selected sites. The model M8, allows positively selected sites with proportion *p*_2_, with ω_2_>1. Likelihood ratio tests (LRT) between nested models (M1a vs. M2a and M7 vs. M8) were conducted by comparing twice the difference in log-likelihood values (2Δ*l*) against a chi-square distribution with two degrees of freedom (d.f.) equal to the difference in the number of parameters between models [26], [27] . If the LRT is significant (p<0.0001), positive selection (ω = dN/dS ratio) is inferred. Bayes Empirical Bayes (BEB) approach (implemented in CODEML) was used to calculate the posterior probabilities (that takes sampling errors into account) of the inferred positively selected sites [28]. Sites with high posterior probabilities (P) coming from the class with ω>1 (P>95%) are inferred to be under positive selection.

### N- and O-glycosylation site analysis

Potential N-glycosylation (Asn-Xaa-Ser/Thr) and O-glycosylation sites were predicted using NetNGlyc 1.0 [29] and NetOGlyc 3.1 [30]. The deduced AA sequences of the second hypervariable region of HRSV-A strains (encompassing AA 210 to the end of the G protein) were compared to those of RSV-A2 and NA1 strains.

### RNA secondary structure and analysis

RNA secondary structures were predicted using the MFOLD web server [31] to compare the relative structural stability of viral RNA (vRNA) and antigenomic RNA (cRNA). Further analysis of vRNA secondary structures was done by using a software tool, ‘mfg’, available at http://www.dbs.umt.edu/research_labs/wrightlab/upload/mfg.html [32]. In a given window size ‘mfg’ folds all nucleotides successively, beginning with each base and predicts the most stable (−ΔG) stem loop structures (SLS), in which that base is unpaired. −ΔG represents the negative free energy. A more negative ΔG value suggests higher stability in the SLS. Mfg calculates the frequency with which a specific base is unpaired in the most stable SLS, giving the result as a “percent unpaired”. A base is called unpaired or paired when present in the loop or in the stem, respectively.

### Genbank nucleotide sequence accession numbers

Representative sequences of RSV-A isolates obtained in this study have been submitted to Genbank under accession numbers JN257682–JN257692 for G-gene and JN257693–JN257703 for F-gene sequences.

## Results

### Clinical specimens and Isolates

Two hundred and three consecutive RSV-positive NPS specimens were identified at PHL between November 2010 and February 2011 from non-ICU, non outbreak patients. Of these, four were positive by the RSV rapid test but were RSV-negative by culture and PCR, and excluded from the study. Among the 199 consecutive RSV isolates included in this study, 47 (23.6%) were obtained from patients reviewed in the emergency room but not hospitalized, 80 (40.2%) collected from hospitalized (non ICU) patients and 21 (10.6%) collected from an ambulatory community setting. There were no data available for the remaining 55 (28%) specimens. RSV-A and B co-infection was identified in 2 samples, which were not evaluated further. One hundred and twelve (55.7%) and 85 (42.3%) of the remaining 197 isolates were identified as subgroup A and B, respectively. Among RSV-A positive specimens, 67 (59.8%) were from infants below 12 months of age, 23 (20.5%) from children 12 to 24 months old, 11 (9.8%) from children 3 to 5 years of age, and 4 (3.6%) were from children of 6 to 10 years of age. Only 2 and 5 isolates were obtained from adults 51 to 66 and 68 to 100 years of age, respectively.

### Molecular analysis of RSV-A strains

**Figure 1:**
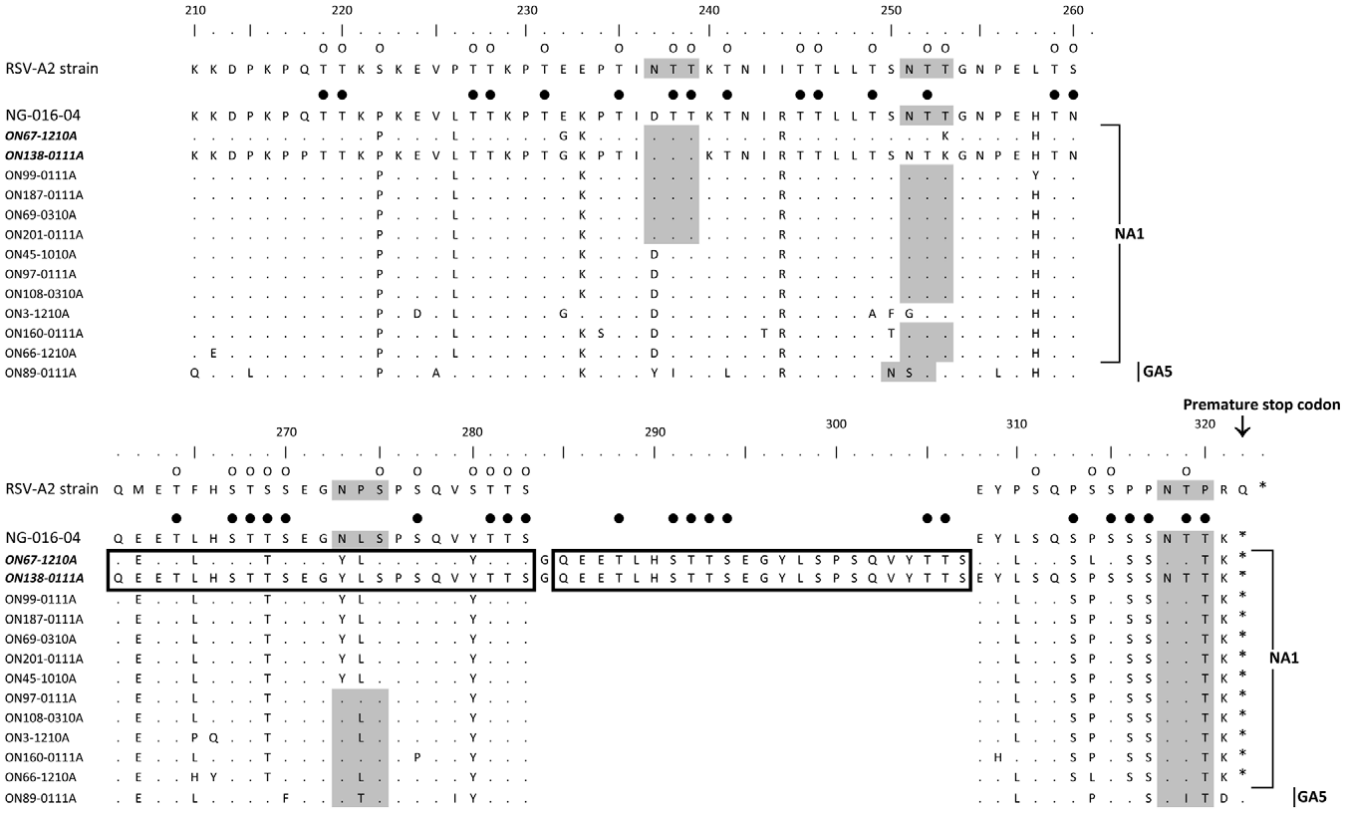
Alignment of deduced amino acid sequence of the G protein of RSV-A strains isolated in Ontario during the 2010–2011 winter season. Alignments are shown relative to the sequences of prototype strain A2 and genotype NA1 strain (AB470478). The AAs shown correspond to positions 201 to 298 of the second hypervariable region of RSV-A strain A2 G protein. The alignment was done by the Clustal W method running within MEGA 5.05. Identical residues are identified as dots. Asterisks indicate the positions of stop codons. The 23 amino acid duplication is enclosed in open boxes. Predicted N-glycosylation sites are shaded in gray. Predicted O-glycosylation sites in RSV-A strain A2 are indicated by small unfilled circles. When compared to the NA1 reference strain, conserved O-glycosylation sites are indicated by black circles.

**Figure 2:**
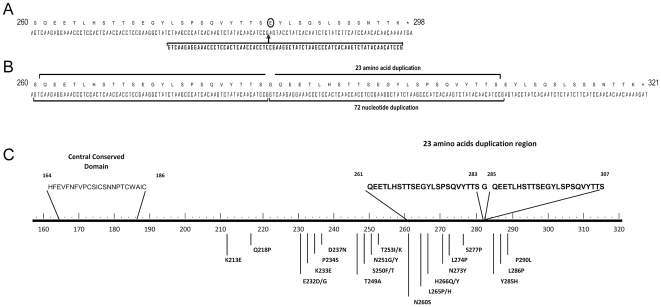
G protein structural features of RSV-A novel genotype, ON1. A) Schematic linear representation of the G protein primary structure of the novel ON1 containing a 72 nucleotide insertion. The amino acid sequence between residues 260 and 298 is shown, highlighting the 72 nucleotide segment that has been duplicated (boldface). The amino acid altered by the insertion is marked with a circle and the point of insertion is indicated by an arrow. B) The 72 nucleotide duplication is indicated by 2 horizontal solid lines below the sequences. The 24 amino acid insertion containing the 23 AA duplication is indicated by 2 horizontal lines above the sequences. Numbers corresponding to AAs indicate that the predicted G polypeptide is lengthened to 321 AAs. C) Graphical representation of the predicted G protein of ON1 with central conserved regions and second variable region identified. Duplicated AA sequences are highlighted in boldface. Positively selected sites are marked with a vertical bar under the line.

By comparing the nucleotide composition and the pattern of mutations among the 112 RSV-A isolates, two major clusters comprising several groups of identical sequences were identified. The alignment of deduced AAs of representative isolates for each group is shown in figure 1. Two previously described genotypes were identified to be currently circulating in Ontario, with 99 (88.4%) belonging to genotype NA1, which is genetically close to GA2 strains [13]. Two isolates were closely related to genotype GA5. A unique observation was the presence of a novel RSV-A genotype (named ON1) including 11 (10%) of the RSV-A isolates which contain a 72 nucleotide duplication (GTCAAGAGGAAACCCTCCACTCAACCACCTCCGAAGGCTATCTAAGCCCATCACAAGTCTATACAACATCCG) in the C-terminal end of the G gene. The duplication starts after residue 850 of the G gene (RSV-A2 prototype numbering) and appears to disrupt the codon “GAG” (residue 850–852) coding for E284, switching it to “GGT” and coding for G284, which is followed by a duplication of 23 AAs (QEETLHSTTSEGYLSPSQVYTTS) spanning positions 261–283 and 285–307 (Figure 2). Although this in-frame duplication does not cause a frame shift, the predicted polypeptide is lengthened by 24 AAs when compared to the reference NA1 genotype. The presence of the G gene duplication was confirmed in the primary specimens of all 10 isolates in which it was detected

The G gene sequence of the Ontario NA1 isolates is closely related to the reference NA1 genotype (AB470478), sharing a high homology of 94.2–98.8% at the nucleotide level and 89.5–98.8% at the amino acid level. However, these ratios dropped to 75.4% and 72.7% at the nucleotide and AA levels, respectively, for ON1 novel RSV-A genotype sequences. Ontario's NA1 and ON1 strains displayed an early stop codon at positions 298 and 322, respectively, when compared to the prototype RSV-A2 strain.

Homology between members of the novel RSV-A ON1 genotype was between 99–100% and 3 unique substitutions, E232G, T253K and P314L (P290L if the duplication is removed), were noted to be specific for this group and not observed in other isolates. Compared to the reference RSV-A2 strain, 16 G gene amino acid substitutions were identified universally among Ontario's NA1 and ON1 strains including S222P, P226L, E233K, N237D, I244R, L258H, M262E, F265L/P/H, S269T, N273Y, P274L, S280Y, P283S, P286L, P289S, S290P/L, P292S, P293S, P296T, and R297K.

The nucleotide sequence of the G gene from the ON1 genotype is translated to a polypeptide of 322 AAs, the largest found so far among RSV-A isolates. The central domain, HFEVFNFVPCSICSNNPTCWAIC , remained conserved among all of Ontario's RSV-A isolates (Figure 2).

Only 2 (1.8%) of the RSV-A isolates, ON/RSV89 and ON/RSV181, were closely related to the GA5 genotype, sharing homology of 94.6%–95.4% at the nucleotide level and 91.9% at the amino acid level with the reference GA5 strain TX67951. They contained several unique mutations including N237Y, S270F, V279I, N297D and H298Q.

### Phylogenetic analysis

**Figure 3:**
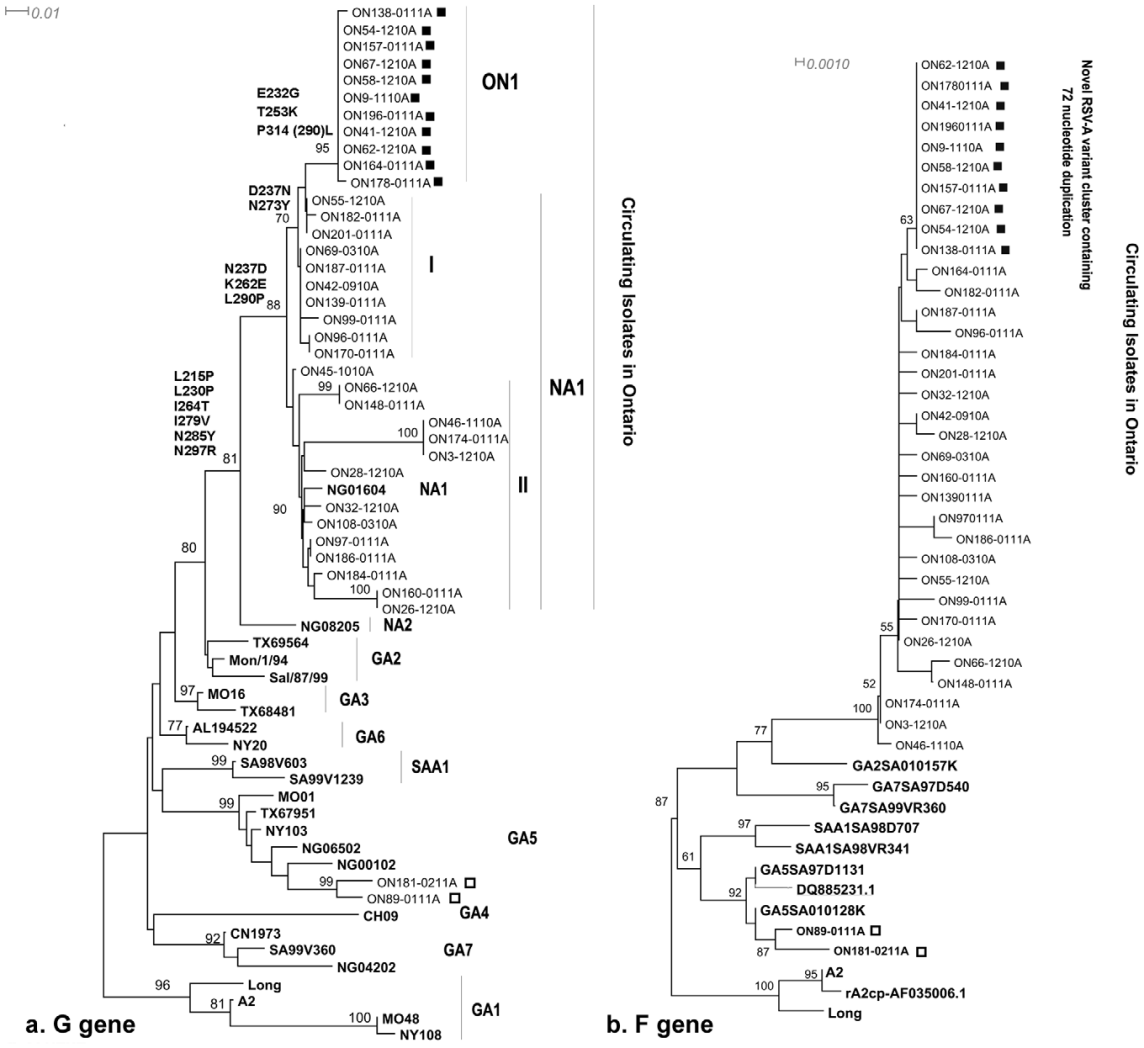
Phylogenetic trees for Ontario RSV-A nucleotide sequences from (a) the second variable region of the G gene and (b) partial F gene sequences. Reference strains representing known genotypes are indicated in bold. Isolates of ON1 genotype circulating in Ontario are indicated by a solid square. Isolates belong to genotype GA5 are marked by an open square. Multiple sequences alignment and phylogenetic trees were constructed using Clustal W and neighbour-joining algorithm running within MEGA 5.05 software. Tree topology was supported by bootstrap analysis with 1000 pseudo replicate datasets. Bootstrap values greater than 50 are shown at the branch nodes. The tree was visualized using Dendroscope software, version 2.2.1.17. The scale bar represents the number of nucleotide substitutions per site between close relatives.

Representative sequences of RSV-A strains circulating in Ontario along with 23 reference strains of RSV-A genotype derived from Genbank were included in the phylogenetic analysis (Figure 3a). Sequencing and phylogenetic analysis shows that the Ontario RSV-A genotypes were classified into three genotypes: NA1, GA5, and a novel genotype, ON1. The two GA5 isolates clustered with a bootstrapping value of 99%. All Ontario NA1 isolates clustered with NA1 genotype (AB470478) with bootstrapping value of 89%. Ontario NA1 isolates were further divided into 2 main clusters, I and II. Several members of these clusters share ≥96% nucleotide similarity and can be designated as individual subtypes of genotype NA1, as proposed by Peret et al [10]. All members of novel ON1 clustered together creating an individual branch with bootstrap value of 94% and p-distance of 0.04. This meets the proposed criteria for a new genotype – a cluster of sequences with bootstrap values of 70%–100% and a p distance of ≤0.07. [12].

Comparing the F gene phylogeny of the study RSV-A isolates based on a 500 nucleotide partial sequence (nucleotides 700–1200; Figure 3b) reveals agreement between the two data sets, with the ON1 genotype again clustering as an individual branch. Nevertheless, due to the lower nucleotide variability, the phylogenetic tree of the F gene region showed less resolution than that of the G gene. Although genetic and antigenic variations occur more frequently in the G protein than F protein, the similarity of both trees confirm the observations drawn from the G gene phylogenetic analysis.

### Glycosylation sites

Different patterns of putative O- and N-glycosylation sites were seen among Ontario isolates. Twenty one and 27 O-glycosylation sites were predicted in ON/RSV89 and ON/RSV181 (GA5 genotype isolates) respectively, whereas 33±2 sites were potentially O-glycosylated (G scores of 0.5–0.8) in Ontario NA1 isolates (Figure 1). All ON1 strains shared a similar profile of O- glycosylation and contained the highest number of O-glycosylation with 37 to 40 predicted sites. The 23AA duplication resulted in duplication of 7 potential O-glycosylation sites. The previously reported AA positions (serine at 267, 270, 275, 283, 287 and threonine at 227, 231, 235, 253, and 282) that are likely to have O-linked side chains were conserved in all Ontario isolates [8]. In addition, AAs 270, 275 and 283 were repeated in the duplicated region of ON1 isolates. By analysing the same region using NetNGlyc 1.0 server [29], four putative N-glycosylation sites (Asn-X-Ser/Thr) were identified among Ontario circulating strains. Only one of four N-glycosylation sites (AA 294 in RSV-A2 strain or AA 318 in the ON1 strains) remains conserved between all Ontario's isolates and RSV-A isolates deposited in Genbank. When compared to the NA1 reference strain (AB470478), two AA substitutions (T253K and N273Y) were observed among ON1 isolates, which led to loss of 2 potential N-glycosylation sites (Figure 1).

### Selective pressure analysis

Relative contributions of selective forces on the evolution of the C-terminal hypervariable region of G-proteins of RSV-A were assessed by measuring the site-specific dN/dS using the PAML program. The average dN/dS ranged from 0.355 to 0.960 among all codon substitution models (Table 1). The M2a and M8 models provide a significantly (p<0.0001) better fit to the dataset as evaluated by the likelihood ratio tests (LRTs) than do their counterpart models, M1a and M7 respectively. Both the M8 and M2a models suggested the presence of positively selected sites with a proportion ranging from 15.99% (*p*_2_ = 0.1599 with ω_2_ = 4.4946) to 17.09% (*p*_2_ = 0.1709 with ω_2_ = 4.2356). A total of nineteen positively selected sites were observed with posterior probability greater than 50% (Table 1).

**Table 1:** Parameter estimates, dN/dS, values of log-Likelihood (*l*), positive selection sites, and Likelihood Ratio Tests (LRT) in the G-gene analysis of RSV-A viruses circulating in Ontario, Canada between November 2010 and February 2011.

Model	Parameter estimates	dN/dS	Log-likelihood(*l*)	Positively selected sites^a^	Model comparison (2Δ*l*, d.f, p)
M1a	ω_0_ = 0ω_1_ = 1.00*p*_0_ = 0.6230(*p*_1_ = 0.3769)	0.377	−872.78	Not allowed	M1a vs. M2a: 19.94d.f = 2**,** p<0.0001
M2a	ω_0_ = 0ω_1_ = 1.00**ω_2_ = 4.2356** *p*_0_ = 0.6330*p*_1_ = 0.1960(***p*** **_2_ = 0.1709**)	0.966	−862.81	K213E, Q218P, E232D/G, K233E, D237N, S250F/T, N251G/Y, T253I/K, L265P/H, H266Q/Y, N273Y, L274P, Y285H	
M7	*p* = 0.0050*q* = 0.0066	0.355	−873.01	Not allowed	M7 vs. M8: 19.56d.f = 2, p<0.0001
M8	*p*_0_ = 0.8400(***p*** **_1_ = 0.1599**)*p* = 0.0325*q* = 0.1281**ω = 4.4946**	0.960	−863.23	K213E, Q218P, E232D/G, K233E, P234S,D237N, T249A,S250F/T,N251G/Y, T253I/K, N260S, L265P/H, H266Q/Y, N273Y, L274P, S277P, Y285H, L286P, P290L	

See Methods for explanation of terms used in parameter estimates column.

Neutral models (M1a, and M7) were compared with their respective alternative (selection) models (M2a and M8), which allow *ω*>1. Model comparison can be calculated using 2Δ*l* = 2 (*l*_1_−*l*_0_)), where *l*_1 = _LRT of alternative model; and *l*_0 = _LRT of null model. Proportion of positively selected sites and their corresponding *ω*-values in M2a and M8 models are in bold. The significant *P* values indicated that all analyses find very strong evidence for the selection model.

a Positively selected sites using Bayes Empirical Bayes analysis [28]. Posterior probability of positively selected sites of M2a model: 50% to 74% (213, 218, 237, 285); 85% to 94% (233, 274); and >95% (232, 250, 251, 253, 265, 266, 273). Posterior probability of positively selected sites of M8 model: 50% to 74% (213, 218, 234, 249, 260, 277, 286); 75% to 84% (237, 285); 85% to 94% (233); and >95% (232, 250, 251, 253, 265, 266, 273, 274, 290).

### Comparison of viral RNA secondary structures

**Figure 4:**
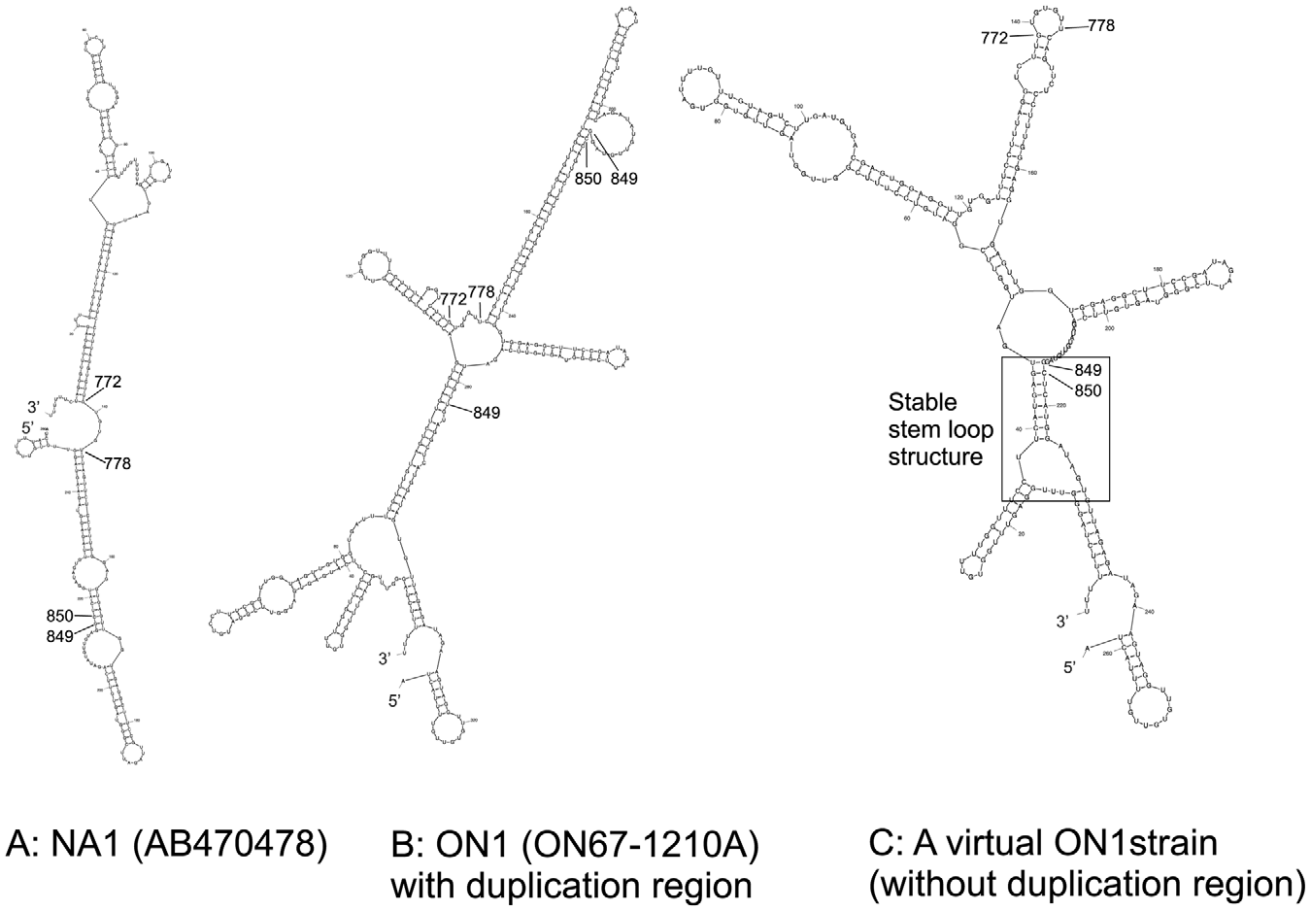
Comparison of predicted viral RNA secondary structures of G gene. Mfold predicted viral RNA secondary structures for G-gene of A. NA1 (AB470478); B. ON1 (ON67-1210A); C. rON1, a virtual ON1 strain without the 72nt duplication. The boxed figure corresponds to the stem loop structure (SLS) implicated in RdRp pausing at nucleotides 849 and 850. ΔG indicates the minimum free energy values (kilocalories per mole).

Three sequences, NA1 (AB470478), ON1 (ON67-1210A) and rON1 (a virtual ON1 strain without the duplication region) were compared to provide insight into the possible mechanisms of the duplication occurrence. When compared to their respective cRNA structures, higher free energies (−ΔG in kilocalories per mole) were observed with the viral RNA secondary structures of the NA1 reference (−47.82 vs. −5.32), ON1 (−80.83 vs. −23.84) and rON1 (−45.57 vs.−12.49) (Figure 4). Different secondary structures were formed for NA1 and rON1 even though they display a sequence similarity of 95.8%. By comparative structural analyses we noticed the formation of a stable stem loop structure (SLS) at nucleotides 849 and 850 in rON1 that was not found in NA1 or ON1. Of note, the duplication in ON1 begins immediately after nucleotide 850. In addition, a 7nt motif (repeat motif), GUGUGUU (nucleotides 772 to 778), was observed in rON1 immediately preceding the first copy of the duplication (Figure 4).

The ‘mfg’ program reports the most stable SLS for a given window size of nucleotides and assumes that each SLS has to be initiated from an unpaired base. Table 2 shows the percent unpaired and free energy for the bases found within the repeat motif (GUGUGUU) and at the base of the stable SLS (nucleotides 849 and 850) (in rON1 only) that precedes the duplication event. As shown in Table 2, nucleotides 849 and 850 exhibited a very low percentage of unpairdness in all simulations, i.e. 6% and 3%, respectively.

**Table 2:** Percentage of nucleotide unpairdness in the region of the 7nt repeat motif at nucleotides 772–778 and 849–850 in RSV-A ON1, rON1 and the reference strain, NA1.

Position	Base	NA1 (AB470478)	rON1, a virtual ON1 strain (without duplication region).				ON1 (ON67-1210A, with duplication region)	
		%unpaired	base	%unpaired	ΔG	fold (SI-B)	base	%unpaired
772	G	100	G	86	−7.3		G	86
773	U	100	U	100	−7.3		U	100
774	G	100	G	100	−7.3		G	100
775	U	100	U	100	−7.3		U	100
776	G	100	G	100	−7.3		G	100
777	U	100	U	80	−7		U	80
778	U	100	U	80	−6.8		U	80
849	G	43	G	6	−0.9	820–849	-	66
850	C	30	C	3	−0.3	821–850	C	50

ΔG Gibbs free energy.

SI-B stem induced backtracking.

## Discussion

In this study we analyzed G and F genes of 112 RSV-A isolates from clinical samples tested during winter 2010–2011 in Ontario, Canada. All sequences were analyzed using various bioinformatics methods in order to better understand the genotype variability, molecular epidemiology and evolutionary adaptability of circulating strains. We documented circulation of two genotypes of NA1 (89% of RSV-A isolates) and GA5 (1.8% of RSV-A isolates) in Ontario during winter 2010–2011. In addition, 11 (10%) of the RSV-A isolates belonged to a novel genotype, ON1, characterized by a 72nt duplication in the C terminal third of the G gene. Our findings differ from an earlier Canadian study, which documented a high prevalence of GA5 and GA7 genotypes among Winnipeg isolates in 2000, with each accounting for 30% of circulating RSV-A isolates at that time [11]. We also observed the trend of co-circulation of several genotypes during one single season as previously documented [3], [10], [33]. Our data suggest that NA1 viruses recently circulating in Ontario are closely related to genotype NA1 which originated in East Asia and spread throughout the world [13]. However, due to the absence of genotyping data from past years in Canada, we were not able to confirm neither the direct migration between continents nor estimate the evolutionary rate for these isolates.

We identified a novel genotype of RSV-A (ON1), with a 72 nucleotide duplication in the C terminal third of the G gene. This duplication resulted in codon disruption and lengthening of the subsequent predicted polypeptide by 24 AAs, including 23 duplicated AAs. Nucleotide duplications have rarely been reported in wild type populations of RSV [15], [34], [35]. Such a large duplication has not been documented in any previously described insertion event; the largest reported to date was a 60nt duplication in RSV-B in Buenos Aires in 1999 [15], [17].

Although the sequence composition among the novel RSV-A ON1 genotype remained conserved, three unique substitutions (E232G, T253K and P314L) were noted to be specific for ON1, and not observed in other Ontario isolates. In addition, two amino acid mutations (T253K and N273Y) which were positively selected sites also resulted in loss of two potential N-glycosylation sites identified previously in the NA1 reference strain (AB470478). Such changes in N-glycosylation sites of the G protein might alter the antigenicity between the genotypes and facilitate binding of circulating antibodies [36].

High homology among wild type NA1 genotypes in Ontario and isolates from Genbank reveals the global distribution of this genotype. On the contrary, the presence of ON1 genotype with a 72 nucleotide insertion in Ontario might suggest the effect of geographical and temporal factors on the genetic evolution of RSV-A, as previously speculated for RSV-B [37]. There is insufficient information to make specific conclusions regarding the exact time of appearance of this new genotype. During preparation of this manuscript, 16 RSV-A isolates collected from April to August, 2011 were studied, and 10 (62.5%) were found to be the novel genotype, ON1. This finding suggests that the ON1 genotype is efficiently replicating and spreading within Ontario, and would be confirmed by genotyping a larger number of isolates over a longer time period. Its apparent rapid spread, and lower prevalence (11%) when first studied during winter 2010-11 suggests that it may have only emerged in Ontario in the months prior to winter 2010-11.

Site-specific evolutionary analysis of the C-terminal hypervariable region of the G protein among Ontario NA1 isolates revealed strong evolutionary selection pressure (dN/dS = 4.4), resulting in 19 positively selected sites compared to the NA1 reference genotype (Table 1). The high range positive selection pressure can be explained by the immunogenic nature of the C-terminal hypervariable region which contains multiple epitopes recognized by both murine monoclonal antibodies and human convalescent sera [38]. Out of 19 positive selection sites among NA1 isolates, K233E, P234S, D237N, L265P/H, N273Y, L274P, L286P and P290L were previously described as escape mutants selected with specific Mabs [34], [39], [40], [41], [42] . When compared to prototype RSV-A2, six AAs (K233E, D237N, N260S, L274P, L286P and P290L) exhibited a “flip-flop” pattern. The substitutions at AAs 274 and 290 resulted in loss of group-specific and strain-specific epitopes [40], [41]. The AA 237 mutation, present in 56% of the Ontario NA1 isolates, suggested the gain of a potential N-glycosylation site. These reverted mutations, particularly in the epitope regions, may decrease the antigen avidity to the current circulating strain specific antibodies [43]. Similar observations of the reverted mutations at 237, 274, 286, and 290 were also reported with Brazilian RSV-A isolates using HyPhy program [43]. Other positively selected sites, E232D/G, S250F/T, N251G/Y, T253I/K, H266Q/Y, and Y285H, are located at antigenic sites, whereas T249A is close to an antigenic site (250–258) [44].

Theoretical mechanisms have been proposed for duplication events during replication and transcription processes [45]–[52]. These studies identified stable RNA secondary structures and direct repeat motifs as sites possibly contributing to the occurrence of duplication events. Similar findings were found in this study, with the observation of SLSs and a 7nt repeat motif in rON1 immediately preceding the duplication region (Figure 4). The previous studies also speculated that the roles of tandem repeats and SLSs in duplication events were independent events. We propose that there may be a mechanism that links both of these features to duplication events, as supported by the structural data (Figure 4) and the ‘mfg’ output (Table 2). Several polymerases have been shown to pause at potential DNA secondary structures formed in large single stranded templates [53]–[57]. Evidence from *in vitro* studies also demonstrates one form of RNA polymerase pausing, called backtracking, where after encountering an obstruction such as a secondary structure, the RNA polymerase reverses its direction and relocates itself upstream [58], [59]. Studies have shown that strong pause sites occur at the base of stems in secondary structures (SS) [55], [60]. We propose that RNA-dependent RNA polymerase (RdRp) pauses and backtracks at bases of stable SLSs such as those at positions 849 and 850 in rON1. This pausing and backtracking of RdRp induced by stable stems is called “Stem-Induced Backtracking” [61]. After the backward slide on the template, RdRp may reinitiate the forward slide on the same template at a particular motif such as GUGUGUU. It should be noted that the GUGUGUU motif precedes the first copy of the duplicated region, suggesting that the GUGUGUU motif might play a role as an anchor site for RdRp. The forward slide of RdRp after backtracking may result in reading of the same region (779–848) that has already been copied and result in duplication of the 72nt region, as seen in ON1. Our findings may enhance understanding of the mechanisms of duplication events in RNA viruses in which secondary structures and direct repeats may facilitate and direct the sliding (backward and forward) of the RdRp along the negative-strand RNA template during replication.

The novel RSV-A genotype (ON1) is of considerable interest because of its 72nt duplication in the G gene C-terminal one-third region, which is the largest duplication described to date in this genus. This area is the target for strain specific neutralizing antibodies and such changes in structure might alter the immunogenicity and pathogenicity of the virus. However, further detailed studies should be undertaken to explore pathogenicity, transmissibility and the replication pattern of this new variant.

The results of this study emphasize the importance of early detection and characterization of newly emerging genotypes. Understanding the effect of the novel RSV-A ON1 genotype 72nt G gene duplication on fitness, virulence and transmissibility could help predict changes in viral phenotype and immunogenicity. It will also provide insight into vaccine potential of the G gene protein. Continued genotyping and molecular epidemiological surveillance of RSV are essential to further understanding RSV evolution and transmission in communities and healthcare settings.

## References

[1] Sullender WM, Mufson MA, Prince GA, Anderson LJ, Wertz GW (1998). Antigenic and genetic diversity among the attachment proteins of group A respiratory syncytial viruses that have caused repeat infections in children.. The Journal of Infectious Diseases.

[2] Beem M (1967). Repeated infections with respiratory syncytial virus.. Journal of Immunology.

[3] Cane PA (2001). Molecular epidemiology of respiratory syncytial virus.. Reviews in Medical Virology.

[4] Anderson LJ, Hierholzer JC, Tsou C, Hendry RM, Fernie BF (1985). Antigenic characterization of respiratory syncytial virus strains with monoclonal antibodies.. The Journal of Infectious Diseases.

[5] Mufson MA, Orvell C, Rafnar B, Norrby E (1985). Two distinct subtypes of human respiratory syncytial virus.. J Gen Virol.

[6] Levine S, Klaiber-Franco R, Paradiso PR (1987). Demonstration that glycoprotein G is the attachment protein of respiratory syncytial virus.. J Gen Virol.

[7] Walsh EE, Hruska J (1983). Monoclonal antibodies to respiratory syncytial virus proteins: Identification of the fusion protein.. J Virol.

[8] Collins PL, Chanock RM, Murphy BR (2001). Fields virology, 4th ed..

[9] Sullender WM (2000). Respiratory syncytial virus genetic and antigenic diversity.. Clin Microbiol Rev.

[10] Peret TC, Hall CB, Schnabel KC, Golub JA, Anderson LJ (1998). Circulation patterns of genetically distinct group A and B strains of human respiratory syncytial virus in a community.. J Gen Virol.

[11] Peret TC, Hall CB, Hammond GW, Piedra PA, Storch GA (2000). Circulation patterns of group A and B human respiratory syncytial virus genotypes in 5 communities in North America.. J Infect Dis.

[12] Venter M, Madhi SA, Tiemessen CT, Schoub BD (2001). Genetic diversity and molecular epidemiology of respiratory syncytial virus over four consecutive seasons in South Africa: Identification of new subgroup A and B genotypes.. J Gen Virol.

[13] Shobugawa Y, Saito R, Sano Y, Zaraket H, Suzuki Y (2009). Emerging genotypes of human respiratory syncytial virus subgroup A among patients in Japan.. J Clin Microb.

[14] Trento A, Viegas M, Galiano M, Videla C, Carballal G (2006). Natural history of human respiratory syncytial virus inferred from phylogenetic analysis of the attachment (G) glycoprotein with a 60-nucleotide duplication.. J Virol.

[15] Trento A, Galiano M, Videla C, Carballal G, Garcia-Barreno B (2003). Major changes in the G protein of human respiratory syncytial virus isolates introduced by a duplication of 60 nucleotides.. J Gen Virol.

[16] Sato M, Saito R, Sakai T, Sano Y, Nishikawa M (2005). Molecular epidemiology of respiratory syncytial virus infections among children with acute respiratory symptoms in a community over three seasons.. J Clin Microb.

[17] Dapat IC, Shobugawa Y, Sano Y, Saito R, Sasaki A (2010). New genotypes within respiratory syncytial virus group B genotype BA in Niigata, Japan.. J Clin Microb.

[18] Johnson PR, Spriggs MK, Olmsted RA, Collins PL (1987). The G glycoprotein of human respiratory syncytial viruses of subgroups A and B: Extensive sequence divergence between antigenically related proteins.. Proc Natl Acad Sci U S A.

[19] Lambert DM (1988). Role of oligosaccharides in the structure and function of respiratory syncytial virus glycoproteins.. Virology.

[20] Garcia-Beato R, Martinez I, Franci C, Real FX, Garcia-Barreno B (1996). Host cell effect upon glycosylation and antigenicity of human respiratory syncytial virus G glycoprotein.. Virology.

[21] Dewhurst-Maridor G, Simonet V, Bornand JE, Nicod LP, Pache JC (2004). Development of a quantitative TaqMan RT-PCR for respiratory syncytial virus.. J Virol Methods.

[22] Sullender WM, Sun L, Anderson LJ (1993). Analysis of respiratory syncytial virus genetic variability with amplified cDNAs.. J Clin Microb.

[23] Parveen S, Sullender WM, Fowler K, Lefkowitz EJ, Kapoor SK (2006). Genetic variability in the G protein gene of group A and B respiratory syncytial viruses from India.. J Clin Microb.

[24] Tamura K, Peterson D, Peterson N, Stecher G, Nei M (2011). MEGA5: Molecular evolutionary genetics analysis using maximum likelihood, evolutionary distance, and maximum parsimony methods.

[25] Kimura M (1980). A simple method for estimating evolutionary rate of base substitutions through comparative studies of nucleotide sequences.. J Mol Evol.

[26] Yang Z (1997). PAML: A program package for phylogenetic analysis by maximum likelihood.. Computer Applications in the Biosciences: CABIOS.

[27] Yang Z (2007). PAML 4: a program package for phylogenetic analysis by maximum likelihood.. Mol Biol Evol.

[28] Yang Z, Wong WS, Nielsen R (2005). Bayes empirical bayes inference of amino acid sites under positive selection.. Mol Biol Evol.

[29] Gupta R, Jung E, Brunak S (2004). Prediction of N-glycosylation sites in human proteins. NetNGlyc 1.0.. http://www.cbs.dtu.dk/services/NetNGlyc/Accessed.

[30] Julenius K, Molgaard A, Gupta R, Brunak S (2005). Prediction, conservation analysis, and structural characterization of mammalian mucin-type O-glycosylation sites.. Glycobiology.

[31] Zuker M, Mathews DM, Turner DH (1999). Algorithms and Thermodynamics for RNA Secondary Structure Prediction: A Practical Guide in RNA Biochemistry and Biotechnology.. Barciszewski J and Clark BFC, editors. NATO ASI Series, Kluwer Academic Publishers, Dordrecht, NL.

[32] Wright BE, Reschke DK, Schmidt KH, Reimers JM, Knight W (2003). Predicting mutation frequencies in stem-loop structures of derepressed genes: implications for evolution.. Mol Microb.

[33] Hall CB, Walsh EE, Schnabel KC, Long CE, McConnochie KM (1990). Occurrence of groups A and B of respiratory syncytial virus over 15 years: Associated epidemiologic and clinical characteristics in hospitalized and ambulatory children.. J Infect Dis.

[34] Garcia O, Martin M, Dopazo J, Arbiza J, Frabasile S (1994). Evolutionary pattern of human respiratory syncytial virus (subgroup A): Cocirculating lineages and correlation of genetic and antigenic changes in the G glycoprotein.. J Virol.

[35] Zlateva KT, Lemey P, Moes E, Vandamme AM, Van Ranst M (2005). Genetic variability and molecular evolution of the human respiratory syncytial virus subgroup B attachment G protein.. J Gen Virol.

[36] Cane PA, Matthews DA, Pringle CR (1994). Analysis of respiratory syncytial virus strain variation in successive epidemics in one city.. J Clin Microb.

[37] Kuroiwa Y, Nagai K, Okita L, Yui I, Kase T (2005). A phylogenetic study of human respiratory syncytial viruses group A and B strains isolated in two cities in Japan from 1980–2002.. J Med Virol.

[38] Melero JA, Garcia-Barreno B, Martinez I, Pringle CR, Cane PA (1997). Antigenic structure, evolution and immunobiology of human respiratory syncytial virus attachment (G) protein.. J Gen Virol.

[39] Cane PA, Pringle CR (1995). Evolution of subgroup A respiratory syncytial virus: Evidence for progressive accumulation of amino acid changes in the attachment protein.. J Virol.

[40] Martinez I, Dopazo J, Melero JA (1997). Antigenic structure of the human respiratory syncytial virus G glycoprotein and relevance of hypermutation events for the generation of antigenic variants.. J Gen Virol.

[41] Lazar I, Canaan A, Weibel C, Kahn JS (2006). Novel mutations in the respiratory syncytial virus G gene identified in viral isolates from a girl with severe combined immune deficiency treated with intravenous immune globulin.. J Clin Virol.

[42] Galiano MC, Palomo C, Videla CM, Arbiza J, Melero JA (2005). Genetic and antigenic variability of human respiratory syncytial virus (groups a and b) isolated over seven consecutive seasons in Argentina (1995 to 2001).. J Clin Microb.

[43] Botosso VF, Zanotto PM, Ueda M, Arruda E, Gilio AE (2009). Positive selection results in frequent reversible amino acid replacements in the G protein gene of human respiratory syncytial virus.. PLoS Pathogens.

[44] Cane PA (1997). Analysis of linear epitopes recognised by the primary human antibody response to a variable region of the attachment (G) protein of respiratory syncytial virus.. J Med Virol.

[45] Kirkegaard K, Baltimore D (1986). The mechanism of RNA recombination in poliovirus.. Cell.

[46] Scott GE, Tarlow O, McCrae MA (1989). Detailed structural analysis of a genome rearrangement in bovine rotavirus.. Virus Res.

[47] Gault E, Schnepf N, Poncet D, Servant A, Teran S (2001). A human rotavirus with rearranged genes 7 and 11 encodes a modified NSP3 protein and suggests an additional mechanism for gene rearrangement.. J Virol.

[48] Matthijnssens J, Rahman M, Van Ranst M (2006). Loop model: mechanism to explain partial gene duplications in segmented dsRNA viruses.. Biochem Biophys Res Commun.

[49] Ballard A, McCrae MA, Desselberger U (1992). Nucleotide sequences of normal and rearranged RNA segments 10 of human rotaviruses.. The J Gen Virol.

[50] Kojima K, Taniguchi K, Kawagishi-Kobayashi M, Matsuno S, Urasawa S (2000). Rearrangement generated in double genes, NSP1 and NSP3, of viable progenies from a human rotavirus strain.. Virus Res.

[51] Wilson V, Taylor P, Desselberger U (1988). Crossover regions in foot-and-mouth disease virus (FMDV) recominants correspond to regions of high local secondary structure.. Arch Virol.

[52] Gorziglia M, Nishikawa K, Fukuhara N (1989). Evidence of duplication and deletion in super short segment 11 of rabbit rotavirus Alabama strain.. Virology.

[53] Kaguni LS, Clayton DA (1982). Template-directed pausing in in vitro DNA synthesis by DNA polymerase a from Drosophila melanogaster embryos.. Proc Natl Acad Sci U S A.

[54] Weaver DT, DePamphilis ML (1982). Specific sequences in native DNA that arrest synthesis by DNA polymerase alpha.. J Biol Chem.

[55] Weaver DT, DePamphilis ML (1984). The role of palindromic and non-palindromic sequences in arresting DNA synthesis in vitro and in vivo.. J Mol Biol.

[56] LaDuca RJ, Fay PJ, Chuang C, McHenry CS, Bambara RA (1983). Site-specific pausing of deoxyribonucleic acid synthesis catalyzed by four forms of Escherichia coli DNA polymerase III.. Biochemistry.

[57] Bedinger P, Munn M, Alberts BM (1986). Sequence-specific pausing during in vitro DNA replication on double-stranded DNA templates.. J Biol Chem.

[58] Galburt EA, Grill SW, Wiedmann A, Lubkowska L, Choy J (2007). Backtracking determines the force sensitivity of RNAP II in a factor-dependent manner.. Nature.

[59] Komissarova N, Kashlev M (1997). Transcriptional arrest: Escherichia coli RNA polymerase translocates backward, leaving the 3′ end of the RNA intact and extruded.. Proc Natl Acad Sci U S A.

[60] Suo Z, Johnson KA (1998). DNA secondary structure effects on DNA synthesis catalyzed by HIV-1 reverse transcriptase.. J Biol Chem.

[61] Shaevitz JW, Abbondanzieri EA, Landick R, Block SM (2003). Backtracking by single RNA polymerase molecules observed at near-base-pair resolution.. Nature.

